# Editorial: Reaction Dynamics Involving Ions, Radicals, Neutral and Excited Species

**DOI:** 10.3389/fchem.2019.00859

**Published:** 2019-12-17

**Authors:** Stefano Falcinelli, Antonio Aguilar, Paolo Tosi, Marzio Rosi

**Affiliations:** ^1^Department of Civil and Environmental Engineering, University of Perugia, Perugia, Italy; ^2^Departament de Ciència de Materials i Química Física, Universitat de Barcelona, Barcelona, Spain; ^3^Department of Physics, University of Trento, Trento, Italy

**Keywords:** ions, radicals, metastable atoms, plasma chemistry, synchrotron radiation, astrochemistry

The aim of this Research Topic is to provide relevant contributions relating to the study of the reactivity of ionic and excited species with atoms, molecules, and radicals of interest in atomic and molecular physics as well as in chemical reaction dynamics. It is well-known that single and multiple ionized species (H^+^, He^+^, ${\text{H}}_{3}^{+}$, HCO^+^, H_3_O^+^, ${\text{He}}_{2}^{2+}$, ${\text{CO}}_{2}^{2+}$, etc.), excited atoms and molecules [e.g., O(^1^D), N(^2^D), H^*^(2s^2^S_1/2_), He^*^(2^1,3^S_0,1_), ${\text{N}}_{2}^{*}$(${\text{A}}^{3}\Sigma_{\text{u}}^{+}$), etc.], and radicals (OH, SH, NH, NH_2_, CH_2_, CH_3_, etc.) play an important role in many chemical systems such as flames, natural plasmas (planetary ionospheres, comet tails, interstellar clouds), and biological environments (e.g., biological tissues damaged when high-energy radiation interacts with a living cell). Such processes have long attracted the attention of the scientific community, as shown by the large number of papers and review articles on this topic, and some specific features make them very interesting from a fundamental point of view in Physical Chemistry and Chemical Physics. However, many applications to important fields like radiation chemistry, plasma physics and chemistry, combustion processes, and the development of laser sources are also possible. In particular, the chemistry of ionic species (both singly and doubly charged ions) is of particular relevance to the conversion of CO_2_ by non-thermal plasma technology. This topic has gained increasing interest in the last few years due to a number of potential advantages, such as working at room temperature with no switch-on inertia or the possibility of obtaining value-added products, like gaseous or liquid fuels, from carbon dioxide with the addition of a hydrogen source (e.g., H_2_O, H_2_, CH_4_, and other hydrocarbons). Such characteristics make this a promising candidate as a technology for the storage of energy from renewable and intermittent sources into chemical energy (see, for instance, Falcinelli et al., 2017; Falcinelli, 2019; Heijkers et al., 2019; and references therein).

This high-quality article collection serves as an opportunity to pay tribute to Davide Bassi, James M. Farrar, and Franco Vecchiocattivi for their relevant contributions over the last 40 years in the fields of atomic and molecular collisions and of reaction dynamics involving ions, radicals, neutral, and excited species. Their biographical notes follow the general description of the articles in this Research Topic.

The articles provide a fairly broad picture of the research conducted nowadays on this scientific topic, both from an experimental and a theoretical point of view. Among the published articles, 20 report experimental data that is then discussed and interpreted with the help of appropriate theoretical models, while nine are purely theoretical studies.

From the experimental point of view, it has to be noted that the molecular beam technique applied to the study of chemical reaction dynamics, coupled with mass spectrometry and spectroscopic techniques (Deckers and Fenn, 1963; Herschbach, 1966; Lee et al., 1969; Scoles et al., 1988; Zare, 2012), constitutes the fundamental scientific background linking all the works presented here.

Almost all of the experimental studies have been carried out using the molecular beam technique. In particular, the two review articles in the collection give an overview of two important applications of this technique: Falcinelli et al. present recent results on the stereo-dynamics of Penning ionization, and Ascenzi et al. report on the possibility of aligning or orienting molecules by collisions in gaseous streams. Furthermore, molecular beams are employed in three different investigations: (i) by Cappelletti et al. in scattering experiments, as a sensitive probe of the intermolecular potentials; (ii) by Rosi et al. in a combined experimental and theoretical work, where the flash pyrolysis of 1-butanol, a bioalcohol belonging to a promising family of biofuels, together with RRKM calculations allowed the characterization of its thermal decomposition; (iii) by Bergeat et al., who in their cross-sectional measurements of C+D_2_ inelastic collisions were able to achieve new insights into the dynamics of collision-induced spin-orbit excitation/relaxation of atomic carbon.

In the study of the characterization and reactive behavior of ionic species, a pair of interesting papers point out the important role of the charge transfer (see Pei and Farrar) and proton transfer (see Canaval et al.) in ion-molecule reactions involving simple cations, as O^+^+CH_4_ and ${\text{NH}}_{4}^{+}$ reacting with various organic molecules [acetone, methyl vinyl ketone, methyl ethyl ketone, and eight monoterpene isomers (C_10_H_16_)], respectively. Moreover, three articles report on the production, characterization, and reactivity of anions (see Shen et al.; Mendes et al.; Avilés-Moreno et al.).

The largest number of experimental works (seven papers) concern the use of synchrotron radiation in studies of the microscopic dynamics of processes involving cationic (Ascenzi et al.; Hrodmarsson et al.; Catone et al.) and dicationic species (Falcinelli et al.). A group of interesting papers concerns the spectroscopic characterization of important molecules: Ferrari et al. focused their work on femtosecond transient absorption spectroscopy of Cobalt tris(acetylacetonate) [Co(AcAc)_3_] in solution; Bolognesi et al. were able to perform core-shell investigation of 2-nitroimidazole. Finally, using synchrotron radiation, Chiarinelli et al. combined photoionization mass spectrometry, the photoelectron-photoion coincidence spectroscopic technique, and computational methods to investigate the fragmentation of metronidazole and misonidazole. In this way, these authors were able to clarify the radiation damage mechanisms of chemotherapeutically active nitroimidazole-derived compounds.

Among the experimental works in this Research Topic, we highlight the paper by Malásková et al., who have compiled a compendium of the reactions of H_3_O^+^ with selected ketones of relevance to breath analysis using the PTR-MS technique (Proton Transfer Reaction Mass Spectrometry). This analytical technique, which is particularly powerful for real-time measurements, is able to provide a valuable database of use to other researchers in the field of breath analysis to aid in the analysis and quantification of trace amounts of ketones in human breath.

Moreover, one paper in the collection is related to the formation of radical and ionic species by plasma-treated organic solvents (see Grande et al.). Another article reports on the characterization of the fluorophores of acridone family compounds by spectrofluorimetric measurements (see Gonzalez-Garcia et al.).

From a theoretical point of view, this Research Topic collects together nine high-quality articles on reaction dynamics. González-Sánchez et al. present a detailed theoretical and computational analysis of the quantum inelastic dynamics involving the lower rotational levels of the MgH^−^ (X^1^Σ^+^) molecular anion in collision with He atoms. De Fazio et al. report on the non-adiabatic, conical-intersection quantum dynamics of the He^+^ + H_2_ → He + H + H^+^ reaction, whereas the quantum dynamics and kinetics of the F + H_2_ and F + D_2_ reactions at low and ultra-low temperatures have been investigated by De Fazio et al.. Furthermore, the chiral rate as a function of temperature between enantiomeric conformations of H_2_O_2_ and Ng (Ng = He, Ne, Ar, Kr, Xe, and Rn) has been studied by de Oliveira Sò et al. at the MP2(full)/aug-cc-pVTZ level of theory through a fully basis set superposition error (BSSE)-corrected potential energy surface.

Molecular dynamics calculations have been performed by Vekeman et al. in investigating graphene layers and proposing them as membranes of subnanometer size suitable for CH_4_/N_2_ separation and gas uptake. Moreover, a procedure adopting an analytic formulation of the potential energy surface (PES), accounting for the dependence of the electrostatic and non-electrostatic components of the intermolecular interaction on the deformation of the monomers, has been used by Lombardi et al. in the generation of a full dimensional PES for the CO + N_2_ system. Furthermore, Xin et al. constructed the first global PES of singlet cyclopropanetrione (C_3_O_3_) at the CCSD(T)/aug-cc-pVTZ//B3LYP/aug-cc-pVTZ level, from which the kinetic stability of a wide range of C_3_O_3_ isomers can be determined by investigating their isomerization and fragmentation pathways.

A deep understanding of the dependence of reaction rates on temperature has been provided by Carvalho-Silva et al., with particular attention to the derivation of the temperature dependence of viscosity. On the other hand, Pietanza et al. investigated the role of the process of dissociative electron attachment from vibrationally excited CO molecules in affecting the whole kinetics of reacting CO under conditions where appreciable concentrations of vibrationally excited states are present.

At the end of this editorial, before the biographical notes of our three colleagues and friends to whom this Research Topic Issue is dedicated, we would like to point out the way we worked together and with all the scientists and colleagues who were glad to share this editorial initiative with us. It is the same attitude, the same lifestyle and behavior that binds Davide, Jim, and Franco: Research and Science carried out within the context of strong human relationships in which ethical, respectful, and friendly behavior is much more important than competition. This is something very special, belonging to Plato's hyperuranion, the realm of ideas that human beings should try to emulate: a beautiful and healthy challenge in our lives.

## Davide Bassi

Davide Bassi (DB) was born in Genoa (Italy) on September 30, 1948. In 1971, he graduated with honors in Physics from the University of Genoa, defending a thesis (under the supervision of Fernando Tommasini and Giacinto Scoles) in which the first evidence of orbiting collisions between atoms was shown. In 1975, he joined the University of Trento, where he established a new Molecular Beam Laboratory. In 1987, he was named full professor of Experimental Physics.

The research activity of DB includes the following topics:

(a) elementary interactions of hydrogen in the gas phase; (b) spectroscopy of atoms, molecules, and clusters; (c) reaction dynamics of ion/neutral systems; (d) ion and neutral beams.

Part of this work has been carried out in cooperation with external laboratories [Department of Chemistry, University of Waterloo (CND), Institut für Ionenphysik, Leopold Franzens Universität, Innsbruck (A), Laboratoire de Physique des Lasers, Universitè Paris Nord, Villetaneuse (F), LURE, Centre Universitaire Paris-Sud, Orsay (F), Dipartimento di Chimica, Università di Perugia (I), Elettra, Gas Phase Beam Line, Trieste (I), Departament de Química Física, and Universitat de Barcelona (E)].

DB has been involved in many research projects supported by the European Commission. In particular, he has been the coordinator of the MCInet European network on the “Generation, stability, and reaction dynamics of Multiply-Charged Ions” (2000–2004). In 2006, he received the SASP Award—in the form of the Erwin Schrödinger Gold Medal—for his contributions to ion-neutral collision studies.

As a spin-off of his “blue sky” research activity, DB developed many industrial applications in the fields of high-vacuum and gas detection technologies. He has been a consultant to private enterprises and participated in the development of long-term joint initiatives between the University of Trento and industrial companies.

From 2004 to 2013, he served as rector of the University of Trento. DB retired from the University of Trento in 2013, at the age of 65, but is still active in the academic arena. Presently, he is a member of the Board of Directors of the Università della Svizzera Italiana (USI) in Lugano (CH) and president of the Ethics Committee of the Italian Institute of Technology (IIT).

## James Martin Farrar

James Martin Farrar was born in Pittsburgh (Pennsylvania, USA) on June 15, 1948. He received his B.A. degree in Chemistry from Washington University in St. Louis in 1970. His first research experience in chemical kinetics was in the laboratory of Professor Joseph Kurz, a classical physical organic chemist. Although Jim was only an undergraduate, Kurz treated him like a colleague, and he found the question “how do chemical reactions actually occur?” to be fascinating. Jim entered the graduate program at the University of Chicago in the Fall of 1970 as a National Science Foundation Graduate Fellow and joined the newly established molecular beam research group of Professor Yuan-Tseh Lee. The group's research was devoted to understanding “where the energy goes in a chemical reaction.” It was during this time in Chicago that Jim met Franco Vecchiocattivi and began to appreciate the importance of ionic interactions and the chemical reactions of ions. The nature of energy redistribution in chemically activated species was also central to the research in Chicago, overlapping with the important work of Davide Bassi on intramolecular vibrational relaxation in optically excited molecules. Following the completion of his degree in June of 1974, Jim spent 2 years in the group of Professor Bruce Mahan learning about the spectroscopy and dynamics of gas-phase ions, topics also of central importance to the research interests of Bassi and Vecchiocattivi. Jim joined the faculty at Rochester in July of 1976, setting up a research laboratory dedicated to applying mass spectrometry and molecular beam methods to the study of gas-phase ion chemistry. He was named an Alfred P. Sloan Fellow in 1981 and was promoted to Associate Professor in 1982. Following his promotion to Professor in 1986, he began a program of research to study the photochemistry of mass-selected solvated metal ions. That work provided evidence of the existence of Rydberg states that serve as precursors for ion-pair production and solvated electron formation. Jim was named a Fellow of the American Physical Society in 1987 and was a Visiting Fellow at JILA at the University of Colorado in 1988. In the early 1990s, Jim and Franco and their colleagues from the University of Perugia initiated a collaboration on collisional ionization that has led to many scientific and personnel exchanges between Rochester and Perugia. Jim served as Chair of the Chemistry Department at Rochester from 1997 to 2000. His most recent research work has applied velocity map imaging methods to the study of ion-radical reactions. The group's research was supported by the U.S. Department of Energy and the National Science Foundation from 1978 to 2017.

## Franco Vecchiocattivi

Franco Vecchiocattivi (FV) was born in Rome (Italy) on July 27, 1945. In 1968, he graduated in Chemistry from the University “La Sapienza” (Rome, Italy), defending a thesis on “Ion-molecule reactions of ethylenimine in the gaseous phase.” After his graduation, he moved to the University of Perugia (Italy), where he joined the first group in Italy that, under the guidance of G. G. Volpi, dealt with the experimental study of the dynamics of chemical reactions with the use of the molecular beam technique.

With the exception of some periods of scientific activity at foreign universities, FV carried out his main teaching and scientific activities at the University of Perugia, where he mainly taught basic courses in general Chemistry for freshmen and more specialized courses for advanced students. At the end of 2015, FV retired from his teaching appointment (full Professor of Chemistry) at the University of Perugia.

In 1973, FV spent half a year at the University of Chicago (USA) in the laboratory of Y. T. Lee, exploiting crossed molecular beam experiments for the study of reactive cross-sections. During that period at the University of Chicago, he collaborated with J. M. Farrar, who was a graduate student at that time.

Upon returning to Perugia from the United States, he started, in collaboration with F. Pirani, a systematic study of the interactions between atoms and simple molecules, integrating high-resolution scattering cross-section measurements with gaseous property data (multiproperty analysis) and producing results of interest for gaseous chemistry.

In 1979 and again in 1981, FV visited the laboratory of V. Kempter at the University of Freiburg (Germany), working on experiments on chemi-ionization reactions at hyperthermal energies.

Returning to Perugia from his visits to Germany, in collaboration with B. G. Brunetti, he designed and built an apparatus to measure total and partial cross-sections of collisional autoionization processes with metastable noble gas atoms in the thermal collision energy range.

In 1990, FV started a fruitful collaboration with J. M. Farrar of the University of Rochester (NY, USA), which continues today. Among his many other scientific collaborations have been those with J. Baudon (University Paris-Nord, France), T. Kasai (University of Osaka, Japan), and A. Aguilar-Navarro (University of Barcelona, Spain).

In recent years, in collaboration with S. Falcinelli, FV started to conduct research at the Gasphase beamline of the ELETTRA Synchrotron of Trieste (Italy), dealing mainly with double photoionization processes of molecules at the threshold.

## Author Contributions

All authors listed have made a substantial, direct and intellectual contribution to the work, participated in writing the manuscript, and approved it for publication.

### Conflict of Interest

The authors declare that the research was conducted in the absence of any commercial or financial relationships that could be construed as a potential conflict of interest.
